# The Human Body

**Published:** 1889-02

**Authors:** 


THE HUMAN BODY; an account of its structure and activities, and the conditions of its healthy working, by H. Newell Martin D. Sc., M. A., M. D., F. R. S Professor of Biology in the Johns Hopkin's University; Fellow of University College, London: Late fellow of Christ's College, Cambridge: fifth edition—revised. New York, Henry Holt and Company 1889. In this work the Author has sought to make the structure and functions of the human organism intelligent to both the general reader and the Medical Student. Not so full or minute in the detai's as usually found in works on Physiology, prepared especially for Medical Students, it yet covers all the leading and essential points which the average Medical Student can be expected to acquire, during a course of Lectures. The Author evinces a thorough acquaintence with the subjects discussed and treats them in such plain and practical way that they may be readily comprehended even by those not possessed of the advantages of higher education. So that the intelligent non professional reader may here find an admirable work for his purposes, while it is also a good text book for the average Student of Medicine.

